# Weighted gene co-expression network analysis identifies CCNA2 as a treatment target of prostate cancer through inhibiting cell cycle

**DOI:** 10.7150/jca.38173

**Published:** 2020-01-01

**Authors:** Rui Yang, Yang Du, Lei Wang, Zhiyuan Chen, Xiuheng Liu

**Affiliations:** Department of Urology, Ren min Hospital of Wuhan University, Wuhan, Hubei, 430060, China

**Keywords:** Weighted gene co-expression network, prostate cancer, prognosis, cell cycle, CCNA2

## Abstract

Prostate cancer is a malignant tumor disease that seriously harms the lives of middle-aged and elderly men. Weighted gene co-expression analysis can be used to construct gene co-expression networks to explore gene sets and genes that are significantly correlated with clinical features. In this study, the transcriptome data of prostate cancer on TCGA was analyzed by weighted gene co-expression network, and the gene with a significant correlation with disease Gleason stage and tumor T stage was identified: CCNA2. CCNA2 was significantly associated with biochemical recurrence, disease-free survival and overall survival rate of prostate cancer. The ability of cancer cell proliferation, invasion and metastasis was decreased after down-regulated expression of CCNA2 in prostate cancer cell lines. Flow cytometry revealed that tumor cells were arrested in the S phase after down-regulated the expression of CCNA2. Taken together, we used WGCNA and obtain a gene CCNA2 which is significantly associated with the prognosis of prostate cancer, which may be an indicator of the prognosis of prostate cancer and a new therapeutic target.

## Introduction

Prostate cancer is a malignant tumor that occurs in middle-aged and elderly men. According to estimates, in the United States, 165000 people were diagnosed and 29000 died because of prostate cancer in 2018. Prostate cancer has already occupied the first place in male patients with new tumors 1; 2. The data from China also shows that prostate cancer ranks seventh among malignant tumors in male patients in 2011. In urban areas, the ranking number rises to sixth place 3. According to statistics from Shanghai, new cases of prostate cancer rank behind lung cancer, stomach cancer, rectal cancer, and liver cancer. The number of new cases in that region will reach 9600 by 2025^4^.

Studies of the prostate cancer transcriptome can help identify molecular subtypes of tumors, key genes in tumors, and discover possible biomarkers 5. The TCGA (The Cancer Genome Atlas) database measured and stored the transcript information and clinical information more than 500 prostate cancer cases. Weighted gene co-expression network analysis (WGCNA) can extract gene co-expression modules and contact it to the clinical feature. It calculates the correlation coefficient value of gene expression and then takes a exponentiation. That opreration make the network meets the scale-free distribution 6. WGCNA also uses soft thresholds and indirect correlations between genes to enhance its biological significance 7. Briefly, by calculating the correlation between the expressions of genes, we could get gene modules which have a high correlation relationship in the genes, and then analysis the correlation between the module and the sample characteristics. It can be considered that WGCNA bridges the gap between sample characteristics and gene expression changes.

In this study, WGCNA was used to analyze hub genes associated with high-stage T stage and tumor Gleason scores in prostate cancer. We also explored the role of key genes in proliferation, invasion, and metastasis of prostate cancer cells.

## Materials and Method

### 1、 Data download and pre-processing

The RNA-seq data of 498 cases of prostate cancer and 52 cases of adjacent tissues were downloaded from the NIH site (https://portal.gdc.cancer.gov). The data format was HTSeq-FPKM. The clinical information of 498 patients with prostate cancer was also downloaded in the format of Clinical BCR XML. The differentially expressed genes in prostate cancer tissues and paracancerous tissues were calculated by R language and limma software package 8. Those genes which P values were less than 0.01 and the absolute value of log2 FC were more than 1 were considered different expression genes. Patients' ID and Gleason scores and pathological T staging were extracted from clinical information.

### 2、Construct a WGCNA network to identify the gene sets associated with tumor Gleason score and pathology T stage

Gene expression matrix, which each row represents a different gene and each column represent a sample, was formed. A clinical feature table that contains Gleason Score and pathology T stage as a numerical value was built. The T stage of each sample was converted into a numerical value (T4: 6, T3b: 5, T3a: 4, T2c: 3, T2b: 2, T2a: 1). First, we excluded the outlier samples by hierarchical clustering. Then we got the best soft threshold according to the value of mean connectivity and the value of scale-independent. The correlation value between every two genes was computed exponentially based on the soft threshold and the results are clustered. The correlation value between gene modules principal component and the clinical feature was calculated. The gene set which had the highest correlation with prostate cancer Gleason and pathological T stage was identified.

### 3、 The analysis of the key gene in the gene module

The STRING database stored information about the interactions about genes and proteins from various dimensions, the database is also convenient for GO (gene ontology) and KEGG (Kyoto Encyclopedia of Genes and Genomes) analysis^9^. Cytoscape is a software for network analysis and editing, its plug ClueGO could easy visualize protein network and find out the hub genes^10^. We extracted the gene names which come from the previous step and put it into the STRING database and obtained the intensity values of the interaction relationship between those genes, the GO analysis results and the KEGG analysis results.We visualized those data with Cytoscape software and R program then we got the top 15 core genes in that gene module.

### 4、CCNA2 expression in prostate cancer

We selected CCNA 2 as the research target after reading the literature. “CamcApp” is an online database containing four prostate cancer research datasets. It is convenient to study the expression and clinical features of CCNA2 gene in prostate cancer 11. “GEPIA” is another online database containing more than 9,000 tumor samples and more than 8,000 normal tissue samples, with sequencing data and clinical data from TCGA and GTEx databases 12. “Oncomine” is another database of sequencing data that contains different tumors 13. We used the data downloaded from the TCGA database to study the expression of CCNA2 in prostate cancer and adjacent tissues, used Tomlins prostate cancer data in Oncomine to study CCNA2 expression in prostate cancer and normal prostate glands. "CamcApp” was used to study the relationship between CCNA2 and biochemical recurrence of prostate cancer, and "GEPIA" was used to study the correlation between CCNA2 and survival of prostate cancer patients.

### 5、Cell culture and transfection

We selected prostate cancer cell lines C42 and PC3 for further study. The cell line was purchased from the China Collection Culture Center (Wuhan, China). Both cell lines were cultured in PRMI- 1640 medium containing 10% fetal bovine serum and 1% penicillin-streptomycin, in the incubator containing 37 ℃, 5% nitrogen dioxide. Fetal bovine serum, penicillin-streptomycin, and PRMI-1640 were purchased from Biyuntian Company (shanghai, China). We used siRNA to downregulate the expression of CCNA2. First, C42 and PC3 cells were seeded at a density of 2*10^5^ per well on a six-well plate, and si-CCNA2 and si-mock were inoculated separately when the cells were grown to about 80%. Transfection reagents, si-CCNA2, and si-mock were purchased from Ribobio Biotech Company (Guangzhou, China). The si-mock was regarded as the NC group, and the si-CCNA2 was regarded as the SI group.

### 6、qPCR detection of prostate cancer in the expression of CCNA2

C42 and PC3 cells inoculated with si-CCNA2 and si-mock were separately gathered, and cells were lysed by adding Trizol reagent, the protein was extracted and the concentration was measured. cDNA of CCNA2 was constructed from retroviral reverse transcriptase. SYBR is the marker to construct real-time quantitative PCR of the reaction system. Reaction conditions: 50 ° C for 2 min, 95 ° C for 10 min; 95 ° C for 30 sec, 60 ° C for 30 sec, 40 cycles. The dissolution curve was plotted and the final data were analyzed by 2^-ΔΔCt^. GAPDH was used as an internal reference. Primer sequence: Homo GAPDH Forward 5'- TCAAGAAGGTGGTGAAGCAGG -3', Reverse: 5'- TCAAAGGTGGAGGAGTGGGT -3', Homo CCNA2 Forward: 5'- CACTCTACACAGTCAGGGGA -3', Reverse: 5'- AGTGTCTCTGGTGGGTTGAG -3 '. qPCR experiments were performed on ABI QuantStudio 6(ABI Life, America), Trizol commercially available from Ambion Inc, SYBR Green Master Mix purchased from the company VAZYME(Nanjing, China).

### 7、Western Blot experiment

The cells in the culture were washed by PBS, and RIPA lysate (Biyuntian Company, Shanghai, China) was added. The supernatant was centrifuged, and the protein concentration was determined by the BCA method. The concentrated gel was run at 80 V and separation gel was run at 120 V constant pressure electrophoresis. After the transference, the membrane was placed in confining liquid for 1 hour at room temperature. The primary antibody was added at 4 °C overnight, and the secondary antibody was added. Finally, the ECL reagent was added, and the gel imaging system was used to photograph. The primary and secondary antibodies were purchased from PROTEINTECH (Wuhan, China), and the gel imaging system was the Bio-rad GelDoc (Bio-rad, America).

### 8、Cell proliferation experiment

The proliferative capacity of C42 and PC3 was determined by the CCK8 assay. The transfected cells were seeded in 96-well plates (1000 cells per well), 100 ul of complete medium was added to each well, 10 ul of CCK8 reagent (Dojindo, Japan) was added after 24 hours, and the absorbance of each well was determined by endpoint method at 450 nm after 2 hours. The microplate reader was the iMark series (Bio-rad, America).

### 9、Cell migration experiment

C42 and PC3 cells were seeded into 6-well plates until cell growth was close to 100%. We use the tip of a sterile pipette tip for scratches. According to the growth of the tumor cells, the washing and photograph operation was repeated. We process the image with ImageJ (NIH, America).

### 10、Cell invasion experiment

The Matrigel (Corning, American) was diluted at different concentrations and inoculated into the Transwell plate chamber. After coagulation, C42 and PC3 cells were inoculated into the upper chamber of the Transwell plate in a serum-free medium at a cell density of 5*10^5^. The lower layer of the Transwell chamber was filled with complete medium containing 10% FBS. After 24 hours, the cells extracted in 4% paraformaldehyde, and stained with 0.1% crystal violet. Tumor cells that pass through Matrigel are colored, images are processed with Image J, and the invasive ability of the cells is assessed based on the number of chromogenic cells.

### 11、flow cytometry

The C42 and PC3 cells after the transfection operation were digested with trypsin and transferred to a centrifuge tube, fixed with 70% ice-ethanol overnight, centrifuged and then added with propidium iodide staining solution. The propidium iodide staining solution was purchased from Biyuntian Company and operated according to the operation manual. The flow cytometer is a BD-FACS Calibur (BS biosciences, American). The results were processed and presented in FlowJo software.

### 12、data processing

The R language and GraphPad prism were used to processes the data. The measurement data is expressed by the mean and standard deviation, and the difference between the groups is measured by the two-sided t-test and chi-square test. All experiments were repeated at least three times. The P-value of less than 0.05 was considered to be statistically different.

## Result

### 1、Pink gene module was significantly associated with Gleason score and pathological T stage of prostate cancer

**Figure 1:**
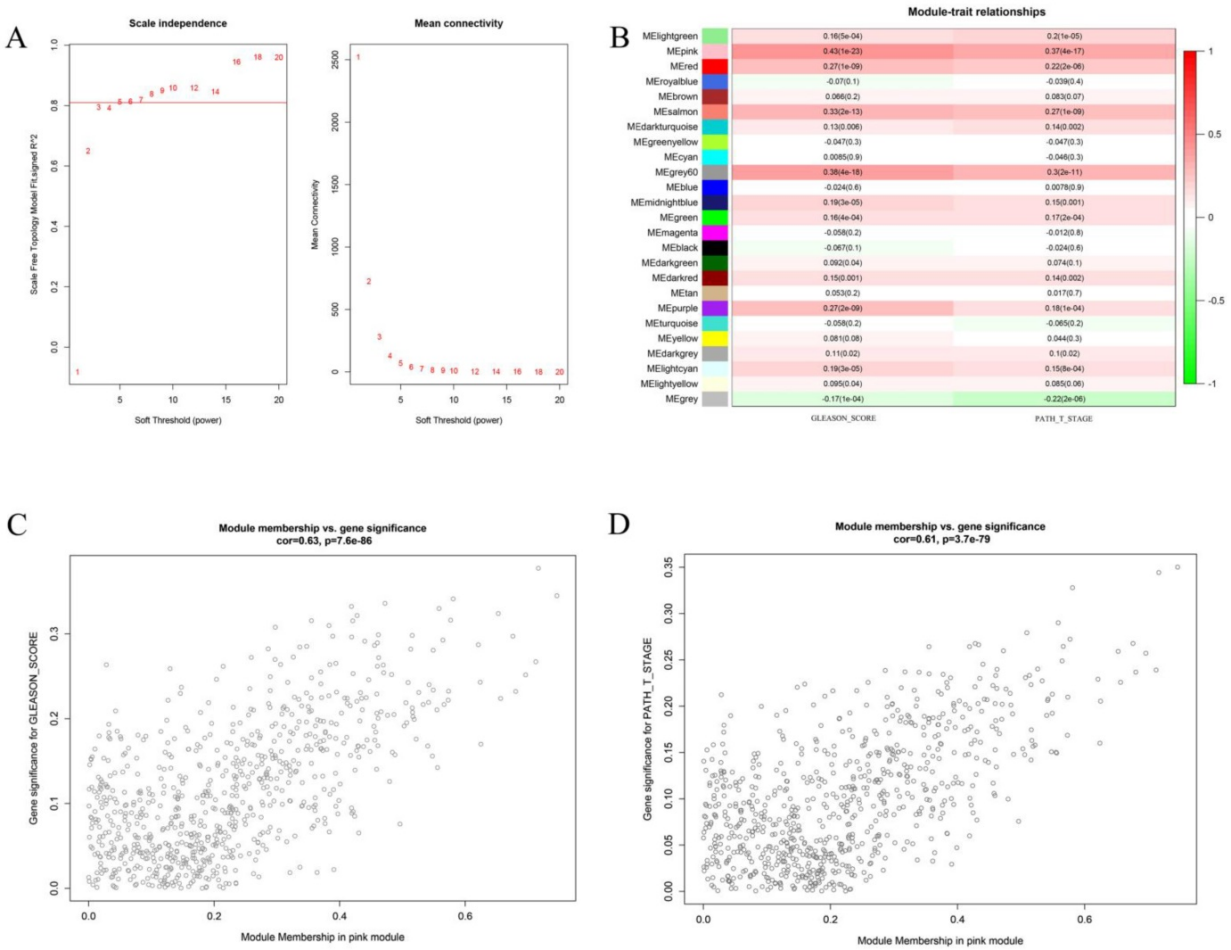
** This figure describes the key steps in obtaining the pink gene module.** Figure 1A shows the determination of soft threshold. Figure 1B shows the correlation between different gene module and the two clinical features. The depth of color represents the value of the correlation, red represents the positive correlation and green represents the negative correlation. Figure 1C shows the correlation analysis between genes in the pink gene module and Gleason score. Figure 1D shows the correlation between the pink gene module and pathological T stage.

We first attempted to identify key gene modules closely related to the two clinical traits of prostate cancer by bioinformatics. Weighted gene co-expression analysis (WGCNA) is a great method to deal with this problem. In the present study, we first compute the soft threshold value. As shown in Figure 1 A, when taking soft threshold at 6, the network could meet the scale-free network according to the value of scale independence and mean connectivity. On this threshold, we continue to carry out further calculations. Figure 1B shows the relationship between different gene modules and the clinical features of Gleason score and pathological T stage. It can be clearly seen that the pink gene had the strongest correlation with the two clinical traits. The correlation coefficients between the pink gene models and the Gleason score was 0.43 (P <0.01), and 0.37 (P <0.01) with pathological T stage. Further analysis shows the Pearson correlation coefficient between the pink genes and Gleason score was 0.63 (P <0.01). The correlation coefficient between the pink genes and the pathological T stage was 0.61 (P < 0.01). It is proved that the increase in Gleason score and the progression of the pathological T stage had great relationship with the pink gene module.

### 2、 CCNA2 was the key genes in the pink gene module

**Figure 2:**
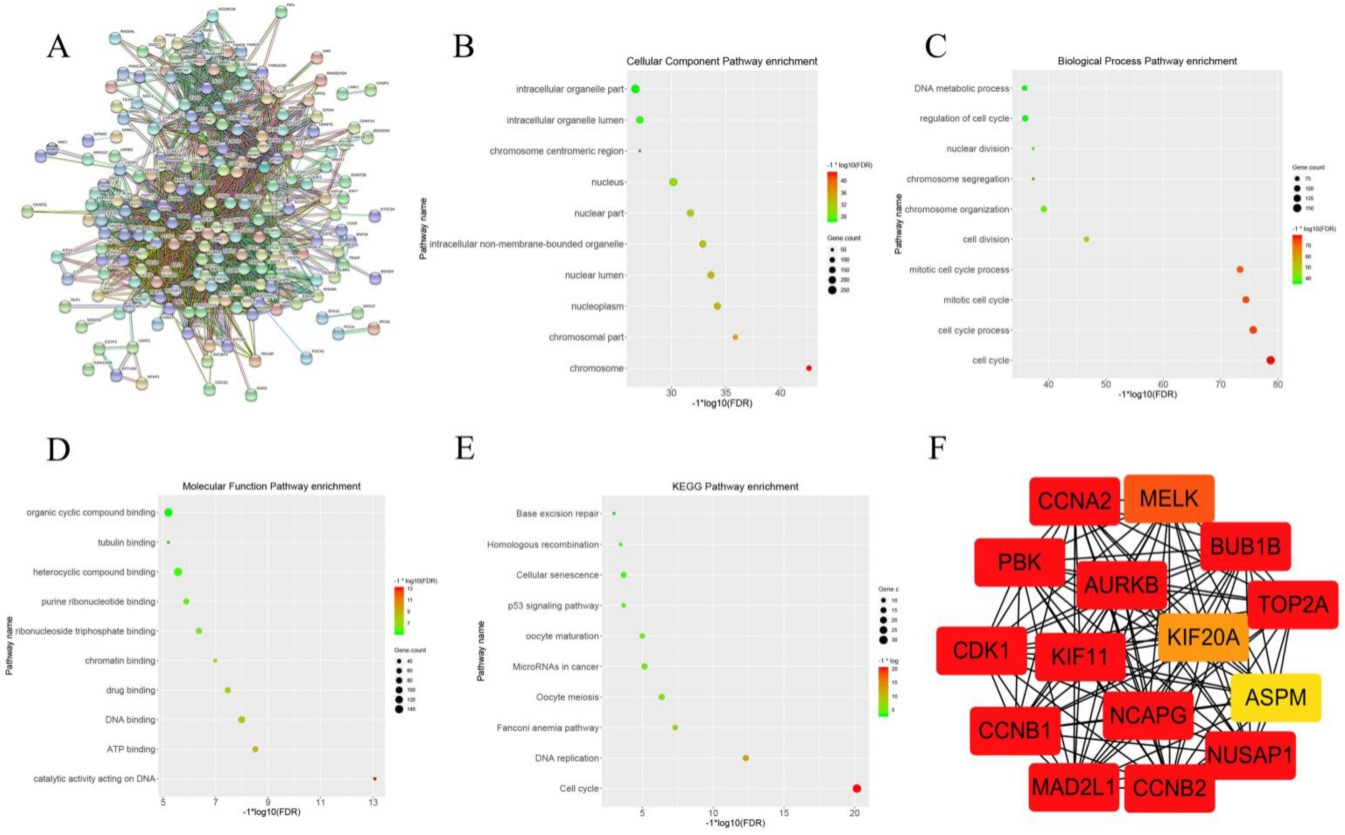
** This graph describes the important steps to obtain the 15 hub genes from the pink gene module.** Figure 2A shows the relationship between pink genes in STRING database. Figure 2B shows the enrichment of pink gene set on Cellular component in GO analysis. Figure 2C shows the enrichment of pink gene set on biology process in GO analysis. Figure 2D shows the enrichment of pink gene set on molecular function in GO analysis. Figure 2E shows the enrichment results of pink gene set on KEGG analysis. Figure 2F shows 15 genes at the core of the pink gene set.

After clarifying the strong correlation between the pink gene module and the Gleason score and the pathological T stage of prostate cancer, we further attempted to determine the key genes in the pink gene module. WGCNA provides a graph-based algorithm to find key genes. However, we believed that the key genes identified by the STRING database based on experimental verification and text mining were more accurate. When we put the gene symbol of the pink gene module into STRING database, we got the picture of Figure 2A. There were 344 nodes in the network, also meant 344 genes. There were 8506 line segments; each line segment meant a relationship between two genes. We also carried out the set of genes to GO analysis and KEGG analysis; cellular component enrichment analysis showed the groups of genes were enrichment in cell organelle part, as shown in Figure 2 B. Biological process enrichment analysis showed that the gene set enrichment appeared on the cell cycle (Figure 2C). Molecular Function enrichment analysis indicated that the set of genes was enriched in the organic cyclic compound binding (Figure 2D). KEGG pathway enrichment analysis suggested that this group of genes was enriched in the cell cycle (Figure 2E). Finally, we use the Cytoscape and its plug ClueGO to visualize the key genes in the pink gene network , the results were shown in Figure 2 F. we have identified 15 genes in the gene pink concentrated at a critical position, named AURKB, CCNB2, CCNA2, MAD2L1, BUB1B, CDK1, CCNB1, KIF, NCAPG, PBK, NUSAP1, TOP2A, MELK, KIF20A and ASPM. By reading the literature and querying the database, we selected CCNA2 as the next research target.

### 3、CCNA2 was highly expressed in prostate cancer and has a significant correlation with prognosis

**Figure 3:**
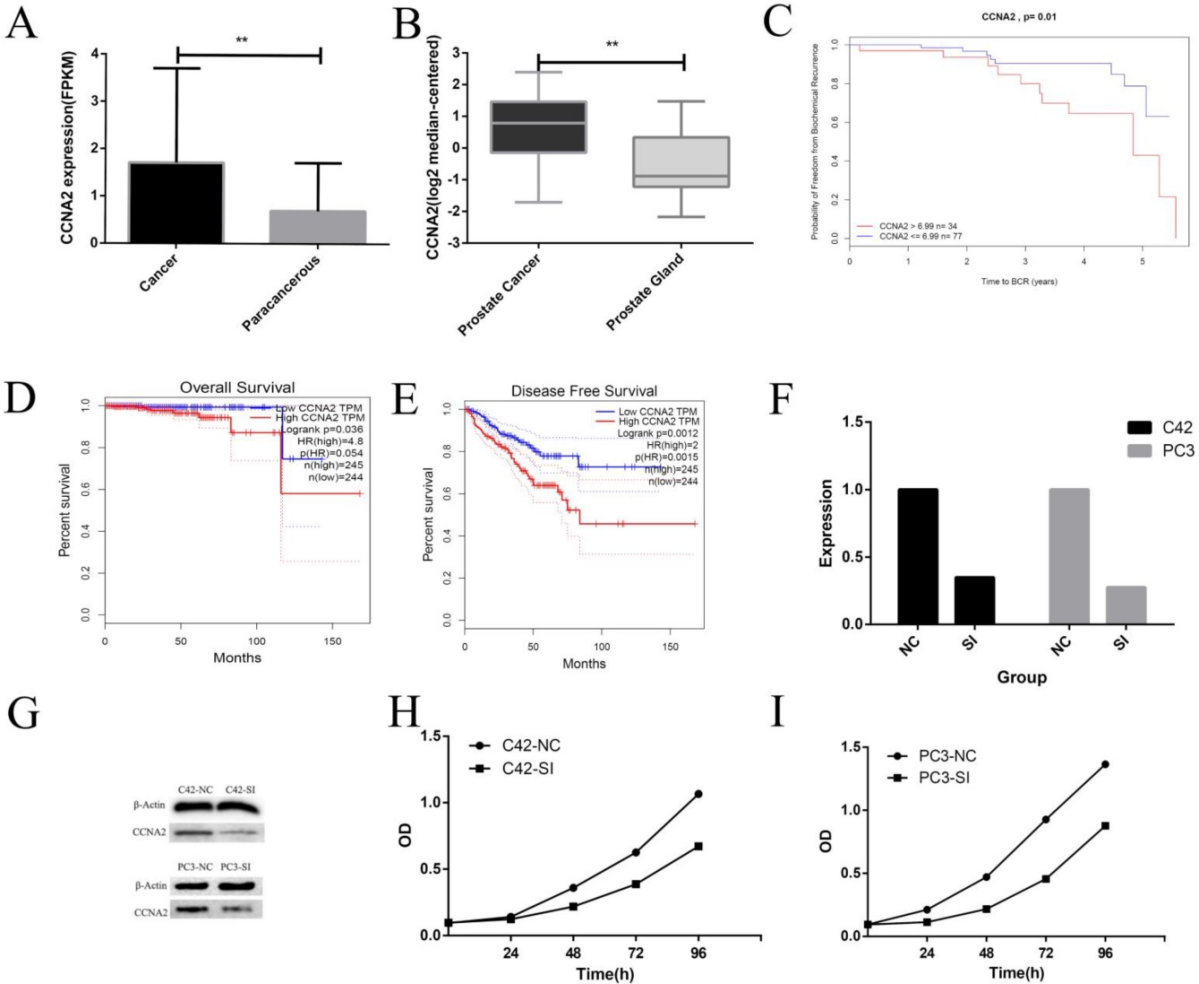
** This figure describes the correlation between CCNA2 and clinical features and some cytological features.** Figure 3A shows the differential expression of CCNA2 between cancer and paracancerous. Figure 3B shows the differential expression of CCNA2 between prostate cancer and normal prostate gland. Figure 3C showed a correlation between CCNA2 and biochemical recurrence rate of prostate cancer. Figure 3D shows the correlation between CCNA2 and overall survival of prostate cancer. Figure 3E shows the correlation between CCNA2 and disease free survival rate in prostate cancer. Figure 3F showed the effect of si-CCNA2 on the down-regulation of CCNA2 mRNA in C42 and PC3 cell lines. Figure 3G showed the effect of si-CCNA2 on the down-regulation of CCNA2 protein in C42 and PC3 cell lines. Figure 3H showed the result on the proliferation rate of C42 cells after down-regulating the expression of CCNA2. Figure 3I showed the result on the proliferation rate of PC3 cells after down-regulation of CCNA2 expression.

We first studied the expression of CCNA2 in prostate cancer and adjacent tissues. The data from TCGA showed that the average expression level of CCNA2 in tumor tissues was 1.87, and the value in adjacent tissues was 0.69. The difference was statistically significant (P < 0.01) (Figure 3 A). Data from the Tomlins prostate cancer dataset in Oncomine showed that the mean expression of CCNA2 in prostate cancer was 0.9, and the expression level in normal glandular prostate was -0.81, the difference was statistically significant (P< 0.01) (Figure 3 B). Subsequently, we used the CamcApp to study the correlation between the expression of CCNA2 and the biochemical recurrence of prostate cancer. It can be seen that there was a significant difference in the biochemical recurrence rate between the CCNA2 high expression group and the low expression group (P = 0.01) (Figure 3C). Then we used the GEPIA database to study the correlation between CCNA2 expression and prognosis of prostate cancer, the results showed the low CCNA2 expression had meaningful relationship with Overall Survival and Disease-Free Survival (Figure 3DE).

### 4. CCNA2 can affect the proliferation, invasion, metastasis and cell cycle of prostate cancer cells

We cultured prostate cancer cell lines C42 and PC3 and down-regulated the expression of CCNA2 by siRNA to observe the effect of CCNA2 on cancer cells. Firstly, we verified the effect of siRNA by real-time fluorescence quantitative PCR, suggesting that our siRNA sequence efficiently silenced CCNA2 in both cell lines. The CCNA2 expression of the C42-SI group was 0.37 times to the C42-NC group, the CCNA2 expression of PC3-SI group becomes 0.22 times of PC3-NC group (Figure 3 F). We next verified the change in the expression of CCNA2 protein by the Western Blot experiment. It can be clearly seen from Figure. 3 G that the expression of CCN2 protein decreased after treatment of cells with siRNA in C42 and PC3. Then, we verified the effect of down-regulating the expression of CCNA2 on cell proliferation by CCK8 assay. In the C42 cell line, it was observed that the expression of CCN2 was down-regulated, and the proliferation of tumor cells was significantly slowed down, and similar results were observed in the PC3 cell line (Figure 3 HI). Scratch test and Transwell experiment showed that after down-regulated the expression of CNA2, the invasion and migration ability of tumor cells decreased, and the difference was statistically significant (P<0.05). Finally, we used flow cytometry to clarify the effect of CCNA2 on cell cycle. The results showed that for both prostate cancer cell lines C42 and PC3, downregulation of cell CCNA2 expression caused cell cycle changes: more cells were stuck in the S phase.

## Discussion

Prostate cancer is a malignant disease that seriously harms men's health, and the molecular mechanism of its pathogenesis is very important. Many studies have attempted to clarify the hub genes which have a great influence on the development and metastasis of prostate cancer from the transcriptome level 14; 15. Weighted genomic co-expression analysis is the development and optimization of co-expression network analysis and has found a number of potential target genes for treating diseases 6. In this study, we used the TCGA database and WGCNA to identify a gene CCNA2 that is closely related to tumor progression and the prognosis of patients with prostate cancer. In addition, we suppressed the expression of CCNA2 in prostate cancer cell lines via siRNA and found that the proliferation, migration and invasion ability of tumor cells were clearly impaired. Flow cytometry experiments indicated that after the down-regulation of CCNA2, tumor cells were enriched in the S phase, suggesting that lowering CCNA2 may affect cell cycle transformation. Our research demonstrates that CCNA 2 may be an important target for the treatment of prostate cancer.

WGCNA is a systematic biological method used to describe the pattern of gene association between different samples 16. It can be used to identify highly synergistically altered gene modules and based on the association between the gene modules and the phenotype of the sample to identify possible therapeutic targets. Compared to the method of focusing only on differentially expressed genes, WGCNA uses thousands of genes to identify the gene modules which make a significant association analysis with the phenotype, thus operation making full use of information. Though this algorithm, the association between thousands of genes and phenotypes is transformed into the association of several gene modules and phenotypes, eliminating the problem of multiple hypothesis testing 17. Many researchers have analyzed their data by using WGCNA and get meaningful results. Ze Zhang et al used WGCNA and found out 12 key genes in head and neck squamous cell carcinoma, which were TIMP2, MIR198, LAMA4, FAM198B, MIR4649, COL5A1, COL1A2, OLFML2B, MMP2, FBN1, ADAM12, and PDGFRB 18. Another study showed that SLC17A7, NTRK2, ABTB1, and ADPRHL1 were key genes in uveal melanoma through using WGCNA and TCGA data 19. In the present study, we used WGCNA to analysis 15,517 genes with differential expression in prostate cancer and paracancerous tissues from 490 samples, and then obtained a pink gene set containing 392 genes with a significant correlation with Gleason stage and pathological T classification of tumors. GO enrichment analysis and KEGG enrichment analysis showed that genes in the pink modules were enriched in cell cycle. We conducted further analysis in that gene module and got the key genes, named AURKB, CCNB2, CCNA2, MAD2L1, BUB1B, CDK1, CCNB, KIF1 1, NCAPG, PBK, NUSAP1, TOP2A, MELK, KIF20A, and ASPM. We selected CCNA2 for further study and found that CCNA2 was significantly associated with biochemical recurrence of prostate cancer. The rate of biochemical recurrence in the CCNA2 low expression group was significantly lower than that in the high expression group (P = 0.01). We also studied the relationship between CCN2 expression and survival rate. The results suggest that the overall survival of CCNA2 low expression group is better than CCNA2 high expression group (P=0.036), and CCNA2 low expression group also kept higher disease-free survival rate than CCNA2 high expression group (P = 0.0012).

CCNA2, also known as Cyclin A2, is a type of cycling protein. CCNA2 gene was first detected in primary liver cancer cells, which appeared in the late G1 and could bind to CDK and CDK2, Respectively; it could affect the G1/ S phase and G2/M phase of the cells 21. It has shown that CCNA2 is associated with multiple tumor prognosis, including kidney cancer, pancreatic cancer, liver cancer, lung cancer, and endometrial cancer 22; 23. In a breast cancer study, CCNA2 was found to be an effective prognostic marker for disease-free survival, overall survival and recurrence-free survival in estrogen receptor-positive breast cancer patients. It has also been found that CCNA2 is significantly associated with tamoxifen resistance in breast cancer patients 24. In a study of liver cancer, researchers found that ZHX2 can repress CCNA2 transcription by binding the promoter regions of CCNA2, thereby inhibiting the proliferation of hepatoma cells 25. Ya qi Gan et al found the expression of CCNA2 was higher in colorectal cancer than the normal colorectum, and reducing the expression of CCNA2 could influence cell cycle, inhibit the proliferation of tumor cells and increase apoptosis^26^. Francesca et al showed that miR-10b may affect tumor cell proliferation and invasion by acting on CCNA2 in breast cancer 27. Ritu et al proved that miR-449a and miR-424 could target CCNA2 and regulate the progress of osteosarcoma 28. Similarly, miR-124, miR-125, miR-219-5p were demonstrated to interact with CCNA2 and affect the growth of different tumor cells 29; 30; 31. In this study, we used WGCNA analysis to obtain the most relevant gene module for tumor progression, while CCNA2 was the key gene in the group of genes. Cytological experiments suggest that down-regulation of CCNA2 can inhibit the proliferation, invasion, and metastasis of prostate cancer cells and affect the cell cycle of tumor cells. In summary, CCNA2 is at the core of tumor development and progression, and many factors might affect the progression of the tumor by affecting the expression of CCNA2. This also suggests that CCNA2 might be a target for the treatment of prostate cancer and will have a high clinical significance.

Taken together, this study combined bioinformatics analysis and cytology experiments and found out the hub gene, which named CCNA2. This study supports the accuracy of bioinformatics methods represented by WGCNA analysis in medical research. However, the deeper mechanism of CCNA2 needs to be strengthened further.

## Conclusion

For the first time, we applied WGCNA analysis to different Gleason scores and different pathological T grades in prostate cancer and found the key genes AURKB, CCNB2, CCNA2, MAD2L1, BUB1B, CDK1, CCNB, KIF1, NCAPG, PBK, NUSAP1, TOP2A, MELK, KIF20A, and ASPM. We studied CCNA2 and found that it has a significant correlation with biochemical recurrence rate and survival rate of prostate cancer. We also verified the role of CCNA2 in prostate cancer cell lines and found that CCNA 2 is associated with tumor cell proliferation, invasion, metastasis, and cell cycle. The study proved CCNA2 is a potential predictor of prostate cancer progression and prognosis and might be an excellent potential therapeutic target.

## Figures and Tables

**Figure 4:**
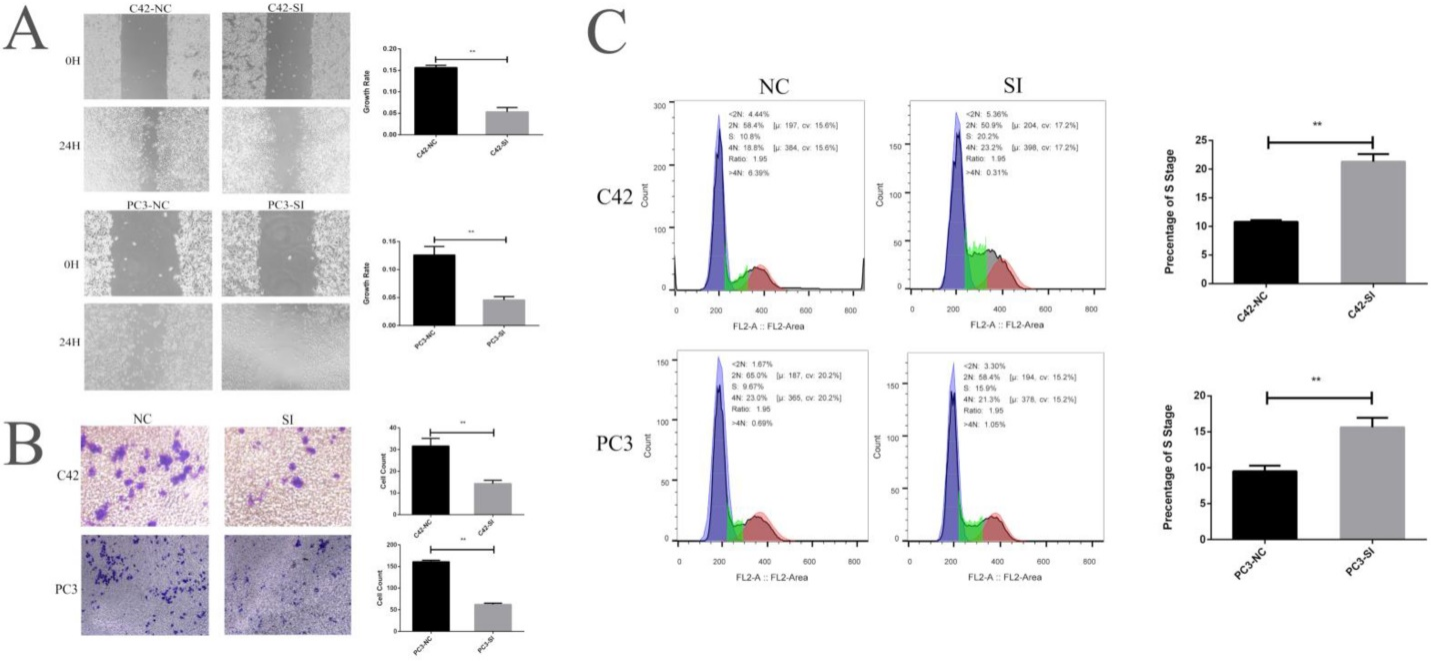
** This figure describes the cytological characteristics of CCNA2 on C42 and PC3.** Figure 4A showed the effect of down-regulation of CCNA2 expression on migration ability of C42 cells and PC3 cells. Figure 4B showed the effect of down-regulation of CCNA2 expression on invasive ability of C42 cells and PC3 cells. Figure 4C showed the effect of down-regulation of CCNA2 expression on the cell cycle of C42 and PC3.
